# A Fairy Chemical Suppresses Retinal Angiogenesis as a HIF Inhibitor

**DOI:** 10.3390/biom10101405

**Published:** 2020-10-04

**Authors:** Deokho Lee, Yukihiro Miwa, Jing Wu, Chiho Shoda, Heonuk Jeong, Hirokazu Kawagishi, Kazuo Tsubota, Toshihide Kurihara

**Affiliations:** 1 Laboratory of Photobiology, Keio University School of Medicine, Tokyo 160-8582, Japan; deokholee@keio.jp (D.L.); yukihiro226@gmail.com (Y.M.); syouda.chiho@nihon-u.ac.jp (C.S.); jeong.h@keio.jp (H.J.); 2 Department of Ophthalmology, Keio University School of Medicine, Tokyo 160-8582, Japan; 3 Animal Eye Care Tokyo Animal Eye Clinic, Tokyo 158-0093, Japan; 4 Research Institute of Green Science and Technology, Shizuoka University, Shizuoka 422-8529, Japan; wu.jing@shizuoka.ac.jp (J.W.); kawagishi.hirokazu@shizuoka.ac.jp (H.K.); 5 Department of Ophthalmology, Nihon University School of Medicine, Tokyo 173-0032, Japan; 6 Graduate School of Integrated Science and Technology, Shizuoka University, Shizuoka 422-8529, Japan; 7 Tsubota Laboratory, Inc., Tokyo 160-0016, Japan

**Keywords:** fairy chemicals, hypoxia-inducible factor, oxygen-induced retinopathy, retinal neovascularization, vascular endothelial growth factor, 2-azahypoxanthine

## Abstract

Neovascular retinal degeneration is a leading cause of blindness in advanced countries. Anti-vascular endothelial growth factor (VEGF) drugs have been used for neovascular retinal diseases; however, anti-VEGF drugs may cause the development of chorioretinal atrophy in chronic therapy as they affect the physiological amount of VEGF needed for retinal homeostasis. Hypoxia-inducible factor (HIF) is a transcription factor inducing VEGF expression under hypoxic and other stress conditions. Previously, we demonstrated that HIF was involved with pathological retinal angiogenesis in murine models of oxygen-induced retinopathy (OIR), and pharmacological HIF inhibition prevented retinal neovascularization by reducing an ectopic amount of VEGF. Along with this, we attempted to find novel effective HIF inhibitors. Compounds originally isolated from mushroom-forming fungi were screened for prospective HIF inhibitors utilizing cell lines of 3T3, ARPE-19 and 661W. A murine OIR model was used to examine the anti-angiogenic effects of the compounds. As a result, 2-azahypoxanthine (AHX) showed an inhibitory effect on HIF activation and suppressed *Vegf* mRNA upregulation under CoCl_2_-induced pseudo-hypoxic conditions. Oral administration of AHX significantly suppressed retinal neovascular tufts in the OIR model. These data suggest that AHX could be a promising anti-angiogenic agent in retinal neovascularization by inhibiting HIF activation.

## 1. Introduction

Vascular diseases of the retina, including diabetic retinopathy, age-related macular degeneration, retinopathy of prematurity and vessel occlusions, are major causes of vision loss worldwide [1,2]. It is well known that the ischemia-mediated overexpression of vascular endothelial growth factor (VEGF) plays a central role in the development of these diseases. The interruption of VEGF signaling has been a good pharmacological target for the treatment of neovascular retinal diseases [3,4]. Thus, anti-VEGF therapies have been widely used to treat pathological angiogenesis in the retina [5]. However, although the small doses used for these diseases and the intravitreal route of the administration may limit systemic side effects, the drugs in chronic therapies can penetrate into blood circulation and alter the systemic or local VEGF levels which may be required for normal vascular and neuronal maintenance [5]. Therefore, many researchers have aimed to develop novel treatments that will help to prevent or suppress pathologic ocular neovascularization without affecting the systemic or local physiological amount of VEGF, or at least minimizing the adverse effects of the current therapies.

Hypoxia-inducible factor (HIF) is a transcriptional factor that regulates various genes for adaptation to cellular hypoxia [6]. Under normoxic conditions, the α subunit of HIF is immediately hydroxylated by prolyl hydroxylase (PHD) and ubiquitinated by von Hippel–Lindau protein. Then, ubiquitinated HIF becomes degraded [6]. Under hypoxic conditions, the activity of PHD decreases resulting in HIF stabilization, then, the stabilized HIF translocates to the nucleus to bind the hypoxia response element (HRE) inducing hypoxia-responsive gene expressions such as VEGF, B-cell lymphoma 2 interacting protein 3 (BNIP3) and phosphoinositide-dependent kinase 1 (PDK1) [7,8]. Therefore, the inhibition of HIF activation under pathologic hypoxic conditions can represent a promising approach to target pathological VEGF expression.

We previously reported that HIF inhibitors, from anti-cancer drugs to natural marine products, suppressed retinal neovascularization and ectopic VEGF expression in murine models of oxygen-induced retinopathy (OIR) and laser-induced choroidal neovascularization (CNV) [9,10,11]. Moreover, another HIF inhibitor, halofuginone—a synthetic derivative of febrifugine isolated from hydrangea—exerted a retinal neuroprotective effect on a murine ischemia–reperfusion model [12].

Mushrooms have been consumed extensively in human’ daily diets and are considered as a delicacy with high nutritional and functional values [13]. They are of interest because of their medicinal attributes [13]. A mushroom, *Hericium erinaceus*, was reported to possess a wide range of therapeutic features such as antioxidant [14], anti-tumorigenic [15] and endoplasmic reticulum stress modulatory activities [16], and hericenones and erinacines found from this fungus are promising candidates for dementia therapies [17]. *Ganoderma lucidum* has been used as a general preventive medicine for thousands of years in eastern Asia. Extracts of *Ganoderma lucidum* exhibited immunomodulatory activity against hypoxia/reoxygenation-mediated neuronal injuries [18]. More than 10,000 species of mushrooms exist in nature [17]; however, most of them have not been studied yet in terms of the bioactive compounds they produce or possess.

In this study, we screened compounds isolated from mushroom-forming fungi for their effect on HIF activity. Furthermore, we explored the therapeutic potential of the strongest candidate, 2-azahyphxanthine (AHX), on a murine model of retinal neovascular degeneration.

## 2. Materials and Methods

### 2.1. Animal

Mice were purchased from CLEA Japan (Tokyo, Japan) and housed in a temperature-controlled environment with free access to food and water under a 12 h light–dark cycle. All animal experimental protocols were approved by the Ethics Committee on Animal Research of the Keio University School of Medicine (approval number #16017-1). Procedures adhered to the ARVO Statement for the Use of Animals in Ophthalmic and Vision Research in accordance with the international standards of animal care and use in the ARRIVE (Animal Research: Reporting in Vivo Experiments) guidelines (http://www.nc3rs.org.uk/arrive-guidelines).

### 2.2. Cell Culture

Murine cell lines of fibroblast 3T3 and cone photoreceptor 661W were cultured in DMEM (Cat #08456-36, Nacalai Tesque, Kyoto, Japan) media supplemented with 10% FBS and 1% streptomycin-penicillin at 37 °C under an atmosphere containing 5% CO_2_. A human cell line of retinal epithelial ARPE-19 was cultured in DMEM/F-12 (Cat #C11330500BT, Gibco, NY, USA) media with the same supplements as above. These cell lines were continuously maintained for in vitro experiments.

### 2.3. Compounds from Mushroom-Forming Fungi

Synthetic AHX and midazole-4-carboxamide (ICA) were kindly supplied by K. Okamoto (Ushio ChemiX Co. Ltd., Shizuoka, Japan). 2-aza-8-oxo-hypoxanthine (AOH) was prepared from AHX by microbial conversion [19]. Erinacine A was isolated from mycelia of *Hericium erinaceus* [20]. Hericenones C, D and E were obtained from fruiting bodies of *Hericium erinaceus* [21]. Grifolin and neogrifolin were obtained from fruiting bodies of *Albatrellus confluens* [22,23]. All samples were stocked in a freezer (−20 °C) for drug screening assays.

### 2.4. Luciferase Assay

A luciferase assay was performed as previously described [12]. Briefly, 3T3, 661W and ARPE-19 cell lines were transfected with a HIF-luciferase reporter gene construct (Cignal Lenti HIF Reporter, Qiagen, Venlo, Netherlands). The HIF-luciferase construct encodes a firefly luciferase gene under the control of the HRE which binds HIF. The cell lines were also co-transfected with a cytomegalovirus-renilla luciferase construct as an internal control and seeded at 1.0 × 10^4^ cells/well/70 µL (3T3 and ARPE-19) or 0.8 × 10^4^ cells/well/70 µL (661W) in a white sterile HTS Transwell-96 receiver plate (Corning, NY, USA). At 24 h of cell stabilization, the cells were treated with CoCl_2_ (200 µM, cobalt (II) chloride hexahydrate, Wako, Saitama, Japan) to activate HIF. To evaluate the inhibitory effects of test compounds (1 mg/mL) against HIF activation, the cells were co-treated with each compound and CoCl_2_. After incubation for 24 h at 37 °C in a 5% CO_2_ incubator, luminescence was measured using a Dual-Luciferase Reporter Assay System (Promega, Madison, WI, USA). 1 mM of Topotecan (Cayman Chemical, Ann Arbor, MI, USA) and 1 mM of doxorubicin (Tokyo Chemical Industry Co., Ltd., Tokyo, Japan) were used for expected HIF inhibitory positive controls.

### 2.5. Quantitative PCR

Total RNA from 661W cells was dissolved in TRI reagent (MRC Global, Cincinnati, OH, USA) and transferred to Econospin columns for RNA extraction and collection (GeneDesign, Osaka, Japan). The columns were washed with buffer RWT and RPE (Qiagen, Hilden, Germany), and the RNA samples were analyzed by ND-2000 spectrophotometer (Thermo Fisher Scientific, DE, USA) to determine the quantity and quality of the samples. RT-PCR was performed using ReverTra Ace qPCR RT Master Mix with gDNA remover (TOYOBO, Osaka, Japan). Real-time PCR was performed using THUNDERBIRD SYBR qPCR Mix (TOYOBO, Osaka, Japan) with a Step One Plus Real-Time PCR system (Applied Biosystems, Waltham, MA, USA). The primers used are listed in Table 1. The fold change between levels of different transcripts was calculated by the ΔΔC*_T_* method.

### 2.6. Western Blotting

Proteins from 661W cells were homogenized in lysis RIPA buffer (Thermo Fischer Scientific, Waltham, MA, USA) containing protease inhibitor cocktail (Roche Diagnostics, Basel, Switzerland). After BCA assay for protein concentration, SDS loading buffer was added to the protein lysates and the lysates were heated at 95 °C for 3 min. The heated lysates were fractionated in 10% SDS-PAGE, transferred to PVDF membranes and then blocked with 5% nonfat dry milk for 1 h. The membranes were incubated with primary antibodies, anti-HIF-1α (1:1000, Cat #36169, Cell Signaling Technology, Danvers, MA, USA), anti-HIF-2α (1:1000, Cat #NB100-122, Novus Biologicals, Centennial, CO, USA) or anti-β-Actin (1:5000, Cat #3700, Cell Signaling Technology, Danvers, MA, USA) at 4 °C overnight. Membranes were washed with TBST several times, and incubated with HRP-conjugated secondary antibodies (1:5000, GE Healthcare, Princeton, NJ, USA) for 2 h at room temperature. Signals were detected using an ECL kit (Ez WestLumi plus, ATTO, Tokyo, Japan). Protein bands were visualized via chemiluminescence (ImageQuant LAS 4000 mini, GE Healthcare, Chicago, IL, USA) and quantified using NIH ImageJ software (National Institutes of Health, Bethesda, MD, USA).

### 2.7. Oxygen-Induced Retinopathy Model and Administration of a Compound from Mushrooms

An oxygen-induced retinopathy (OIR) model was produced as previously described [11]. Briefly, postnatal day 8 (P8) mice (six male and five female pups) were exposed to 85% O_2_ for 72 h in an oxygen supply chamber with their nursing mothers. After oxygen exposure, the mice were placed back in room air until P17. The pups received oral administration of AHX (300 mg/kg/day) or ultrapure water as a vehicle once a day from P12 to P16. For the oral administration, a thin tube filled with AHX or ultrapure water was inserted into the pups’ mouths and AHX or ultrapure water was gently administered toward their esophagi. At P17, the mice were sacrificed, and the eyes were enucleated. The enucleated eyes were fixed for 15 min in 4% paraformaldehyde (PFA) solution. Retinal whole mounts were post-fixed in 4% PFA for 1 h. After several times of washing with PBS, the retinal whole mounts were stained with isolectin GS-IB4 from *Griffonia simplicifolia* conjugated with Alexa Fluor 594 (Invitrogen, Carlsbad, CA, USA) at 4 °C for 3 days. After encapsulation, retinal vessels in the retinal whole mounts were observed with a fluorescence microscope (BZ-9000, KEYENCE, Osaka, Japan). Photographs of the retinal whole mounts were obtained at 10 x magnification and merged into a single using BZ-II Analyzer (KEYENCE, Osaka, Japan). The number of pixels in neovascular tufts and vaso-obliterations was measured using the lasso and magic wand tools in Photoshop (Adobe, San Jose, CA, USA), of which method is the most widely used [24].

### 2.8. Statistical Analysis

Analyses of data from all experiments were performed with GraphPad Prism 5 (GraphPad Software, San Diego, CA, USA). Statistical significance was calculated using a two-tailed Student’s *t*-test or one-way ANOVA followed by a Bonferroni post hoc test. *p*-values of less than 0.05 were considered statistically significant.

## 3. Results

### 3.1. AHX Showed an Inhibitory Effect on HIF Activity

**Figure A1:**
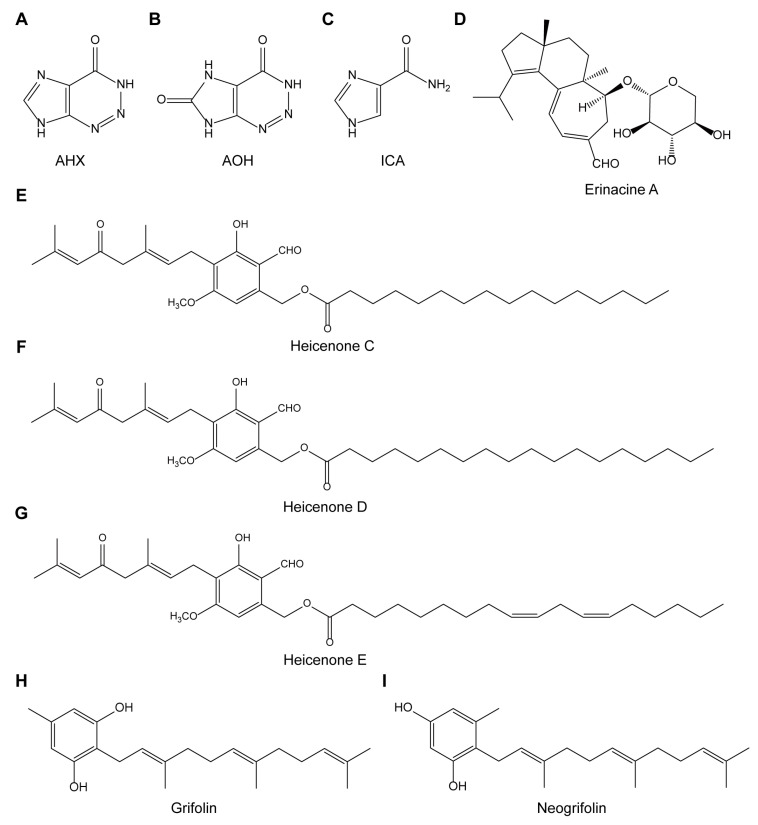
Structural formulas of nine compounds from mushroom-forming fungi. Structural formulas of (**A**) AHX, (**B**) AOH, (**C**) ICA, (**D**) erinacine A, (**E**) heicenone C, (**F**) heicenone D, (**G**) heicenone E, (**H**) grifolin and (**I**) neogrifolin are depicted.

Nine compounds originally from mushroom-forming fungi were obtained (see the “Materials and Methods” and Appendix A, Figure A1) and screened for HIF inhibitory activity via HIF luciferase assay (Table 2). 200 µM of CoCl_2_ was used to induce HIF activation, and 1 mM of topotecan and 1 mM of doxorubicin were used as expected positive controls for HIF inhibition. A relatively high dose of each compound (1 mg/mL) was chosen for gross examination. At the first screening, 3T3 cell line was used, as this cell line has been widely used for the general understanding of roles of HIF, and the development of a HIF-luciferase reporter stable cell line is easily available [25]. At the first screening in 3T3 cells, AHX and AOH showed significant inhibitory effects on HIF activation induced by CoCl_2_ (Table 2). We proceeded to the next stage by using in vitro cell models for ophthalmic drug development [26]. Thus, ARPE-19 cell line (human retinal pigmented epithelium cells) and 661W cell line (mouse immortalized cone photoreceptor cells with some features of retinal ganglion precursor-like cells) were chosen [27,28]. Through the second screening in ARPE-19 cells, we found that AHX only showed a statistically significant HIF inhibitory effect. At the third final screening in 661W cells, the HIF inhibitory effect of AHX was also confirmed.

**Figure 1:**
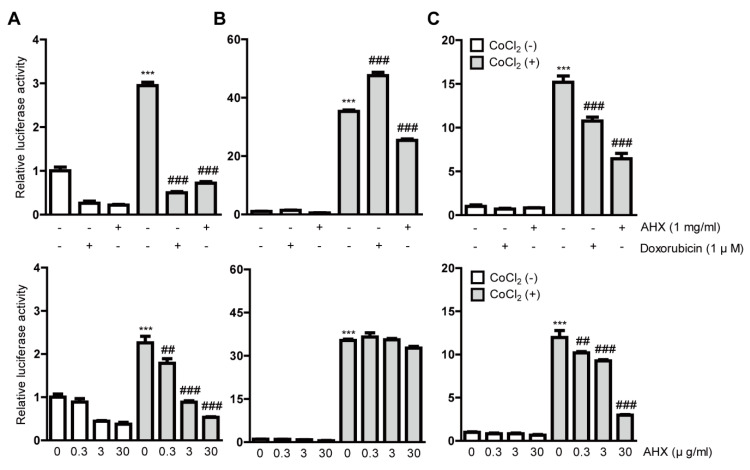
Inhibitory effects of AHX isolated from mushrooms on HIF activation. Quantitative analyses of the HIF-reporter luciferase assays using (**A**) 3T3, (**B**) ARPE-19 and (**C**) 661W cells (*n* = 3–6 per group) showed that AHX inhibited HIF activity induced by 200 µM CoCl_2_ in all of the cell lines. *** *p* < 0.001, ^##^ *p* < 0.01, ^###^ *p* < 0.001 compared with no treatment and 200 µM CoCl_2_ treatment, respectively. Bar graphs are presented as the mean with the ± standard error of the mean. The data were analyzed using one-way ANOVA followed by a Bonferroni post hoc test.

**Figure 2:**
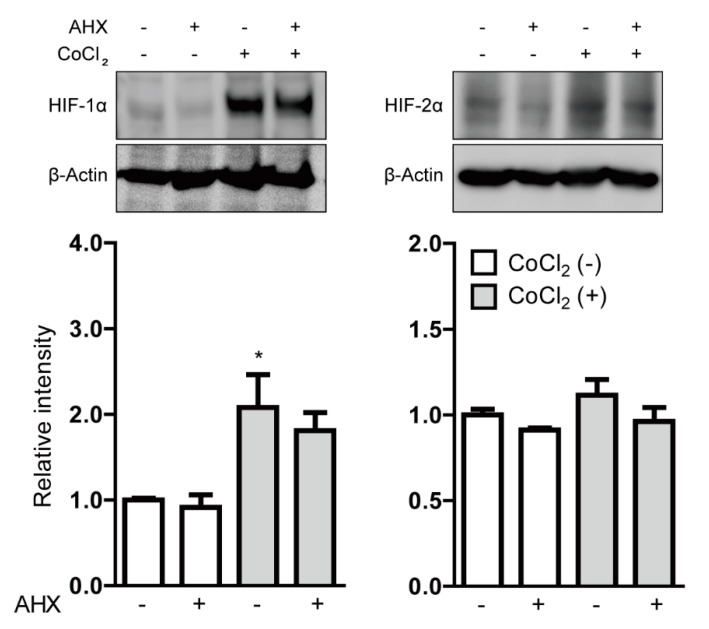
The inhibitory effect of AHX on HIF stabilization. The representative immunoblots and quantitative analyses (*n* = 4 per group) for HIF-1α, HIF-2α and β-Actin showed that only HIF-1α was stabilized in 661W cells under 200 µM CoCl_2_-induced pseudo-hypoxic conditions. Note that AHX did not significantly decrease the stabilized HIF-1α expression. * *p* < 0.05 compared with no treatment. Bar graphs are presented as the mean with the ± standard error of the mean. The data were analyzed using one-way ANOVA followed by a Bonferroni post hoc test.

After the gross screenings, we treated all the cell lines with 1 mg/mL AHX again to clarify the inhibitory effects of HIF and also examined the dose dependency of HIF inhibition by AHX treatment (Figure 1). AHX treatment at 0.3, 3 and 30 µg/mL significantly inhibited the HIF activity in a dose-dependent manner in 3T3 and 661W cells (Figure 1A,C). In contrast, the inhibitory effect of HIF activity in low-dose AHX treatment was not observed in ARPE-19 cells (Figure 1B). Next, we examined whether AHX had an inhibitory effect on HIF stabilization at protein levels (Figure 2). Even though HIF-2α expression was not changed, HIF-1α expression was significantly stabilized in 661W cells after 6 h incubation of 200 µM CoCl_2_. However, the stabilized HIF-1α was not significantly reduced by 1 mg/mL AHX treatment (Figure 2) although the inhibition of HIF activation by AHX was constantly observed (Figure 1C). These results indicate that AHX might have an inhibitory effect on the DNA binding of HIF-1α, suppressing the induction of HIF-1α target gene expressions rather than inhibiting HIF-1α stabilization.

### 3.2. Hypoxia-Responsive Gene Expressions Were Suppressed by AHX Treatment under CoCl_2_-Induced Hypoxic Condition

**Figure 3:**
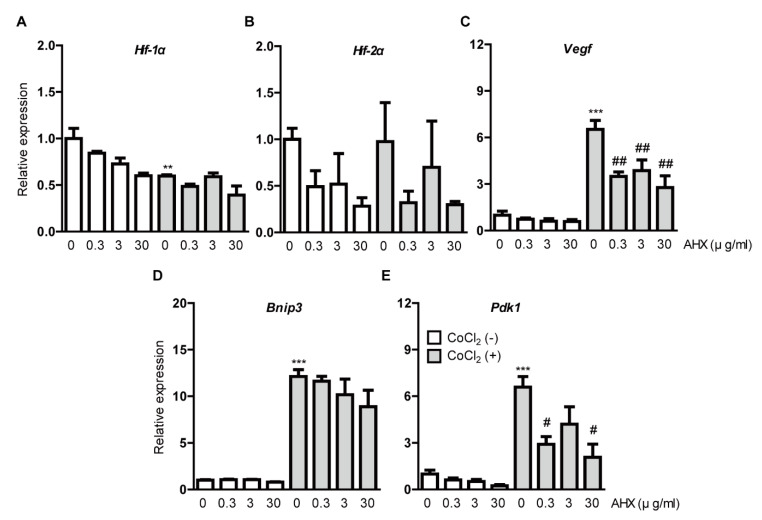
Suppression of hypoxia-responsive gene expressions by AHX treatment. Relative fold changes in (**A**) *Hif-1α*, (**B**) *Hif-2α,* (**C**) *Vegf*, (**D**) *Bnip3* and (**E**) *Pdk1* mRNA expressions were shown after 6 h of AHX treatment under 200 µM CoCl_2_-induced pseudo-hypoxic condition in 661W cells by quantitative PCR analyses (*n* = 3–6 per group). The upregulated *Vegf* and *Pdk1* mRNA expressions were suppressed by AHX treatment. ** *p* < 0.01, *** *p* < 0.001, ^#^ *p* < 0.05, ^##^ *p* < 0.01 compared with no treatment and CoCl_2_ treatment, respectively. Bar graphs are presented as the mean with the ± standard error of the mean. The data were analyzed using one-way ANOVA followed by a Bonferroni post hoc test.

We examined whether AHX suppresses HIF downstream-target hypoxia-responsive gene expressions under CoCl_2_-induced hypoxic conditions (Figure 3). After 6 h incubation of 200 µM CoCl_2_ in 661W cells, the downregulation of *Hif-1*α mRNA expression was detected due to a negative feedback from the post-translational HIF-1α protein modification (Figure 3A) [9,29]. We could not detect alteration of *Hif-2*α mRNA expression (Figure 3B). CoCl_2_ induced the upregulation of *Vegf*, *Bnip3* and *Pdk1* mRNA expressions as a result of HIF-1α activation (Figure 3C–E) [9,29]. The upregulated mRNA expressions, especially *Vegf* and *Pdk1*, were suppressed by AHX treatment (Figure 3C,E). Although the upregulated *Bnip3* mRNA expression was not significantly reduced by AHX treatment, its expression showed a decreasing tendency in a dose-dependent manner (Figure 3D). Under a normal condition, *Hif-1*α and *Hif-2*α mRNA expressions showed a decreasing tendency by AHX treatment but changes in their expressions were not dramatic (Figure 3A,B). Moreover, *Vegf*, *Bnip3* and *Pdk1* mRNA expressions were not significantly altered by AHX treatment (Figure 3C–E).

### 3.3. Neovascularization in a Murine OIR Model Was Suppressed by AHX Administration

**Figure 4:**
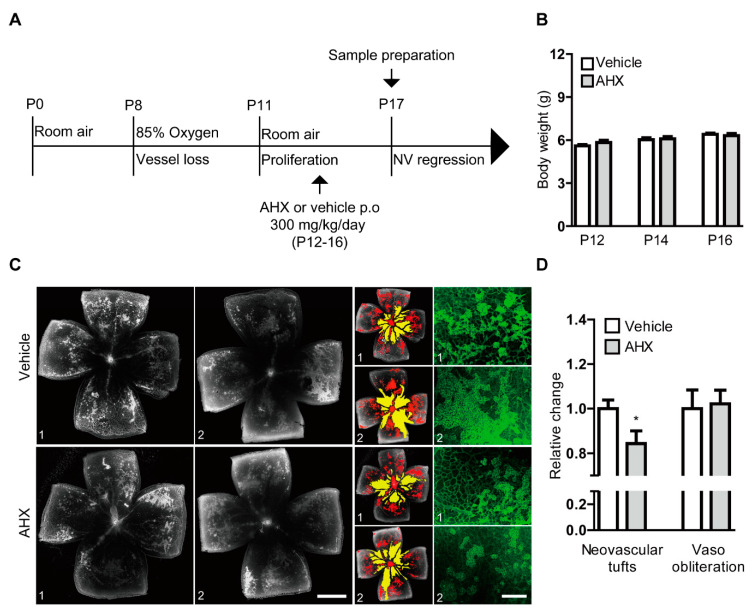
Suppression of neovascularization by oral administration of AHX. (**A**) The schematic illustration shows the murine OIR model procedure and the oral administration of the AHX or vehicle to mice. (**B**) Quantitative analyses for body weights of the mice (*n* = 5–6 per group) showed that the average body weights of the AHX-administered group (three male and three female pups) did not significantly differ from those of the vehicle group (three male and two female pups) during the administration period. (**C**) Representative images of retinal vascular whole mount (vehicle 1, 2 and AHX 1, 2) staining with isolectin B4 (neovascular tufts: red and vaso-obliteration: yellow), and higher-magnification images (green) for neovascular tufts. Scale bars are 1000 and 200 µm, respectively. (**D**) Quantitative analyses showed that areas of neovascular tufts (red) were suppressed in the AHX-administered group (*n* = 6) in comparison with those in the vehicle-administered group (*n* = 5), while no significant difference in vaso-obliteration (yellow) was observed between the groups. * *p* < 0.05. Bar graphs were presented as mean with ± standard error of the mean. The data were analyzed using a two-tailed Student’s *t*-test.

To assess the therapeutic effect of AHX on anti-retinal neovascularization, we orally administered AHX to OIR mice and analyzed neovascular tufts and vaso-obliteration in retinal whole mounts (Figure 4). AHX was administered according to the schedule depicted in Figure 4A. There was no dramatic difference in body weights between the two groups throughout the administration period (Figure 4B). The AHX-administered retinas showed a significant decrease in neovascular tufts compared with the vehicle (water)-administered retinas, while no significant difference was observed in vaso-obliteration between the groups (Figure 4C,D). With higher-magnification of retinal images, decreases in neovascular tufts could also be observed in the AHX-administered retinas (Figure 4C).

## 4. Discussion

AHX is one of the “fairy chemicals” (FCs) that were discovered to be a causing principle of the mysterious natural phenomenon “fairy rings” [30,31]. Fairy rings are arcs of plant growth occurring on the floor of grasslands surrounded by fungi, and these rings are formed by an interaction between grasses and fungi [32]. AHX and ICA were found from a culture broth of the fungus *Lepista sordida*, and AOH was found as a metabolite of AHX in plants [31,33]. Recently, these compounds have been classified as FCs [31]. Among the FCs, AHX showed a dramatic growth-enhancing activity toward all of the plants tested, including crops such as rice and wheat, regardless of the families to which they belong [34,35]. Therefore, AHX has been recommended for its promising practical uses in agriculture [33]. Although AHX has been reported to be a photolytic degradation product from an anti-tumor drug in mammalian studies [36,37,38], there has been no report on the therapeutic effects of AHX in ophthalmology. This is the first report to apply AHX to anti-ocular degeneration.

HIF plays an important role in maintaining cellular homeostasis in response to changes in oxygen status [6,7,8]. Angiogenesis is one of the most well-known hypoxic responses and is mediated by HIF [39]. Under pathological hypoxic conditions in the retina, HIF can be activated and lead to abnormal increases in its downstream gene expressions, especially VEGF, finally causing retinal neovascularization. Previously, pharmacological intervention such as the administration of digoxin [40], aloe-emodin [41], topotecan or doxorubicin [11], acriflavine [42] and fish-derived products [10], suppressed pathological retinal angiogenesis by inhibiting HIF activity and its downstream pathways including VEGF. In this study, we found that AHX showed an inhibitory effect on HIF activity and a suppressive effect on *Vegf* mRNA expression under CoCl_2_-induced hypoxic conditions. Furthermore, AHX suppressed retinal neovascularization in the murine OIR model. In fact, more data are required to understand the in vivo mode of action regarding the reduction in VEGF levels in the retina by AHX administration, which needs to be further studied. Nonetheless, we suggest that HIF/VEGF inhibition can be a promising approach for managing ocular neovascularization.

The development of ocular neovascularization involves complex pathological mechanisms. There is an involvement of multiple interlinked functional and structural alterations by the abnormal crosstalk between retinal neurons, glial cells (astrocytes and Müller glia) and vasculatures [43]. In the central nervous system, neurons (alongside glial cells) act as oxygen sensors and vascular regulators by interacting with vasculatures [44], and impairment of their crosstalk affects neurovascular homeostasis, as observed in neurodegenerative disorders such as Alzheimer’s and Parkinson’s diseases [44,45]. When it comes to the eye, a previous study indicated that the partial actions of retina-derived and astrocyte-derived VEGF could control the proliferation and migration of astrocytes, which is important for retinal vascular development [46]. In addition, the activation of Hif-1α in retinal neurons or Hif-2α in Müller glia played critical roles in retinal vascular diseases [46]. It is still unclear how different cell types (neurons, glia and vascular cells) in the retina respond to hypoxia and interact with each other to induce retinal neovascularization. In our OIR model, we previously demonstrated no alteration in *Hif-2α* mRNA expression and a slight increase in Hif-2α protein expression in the retina while *Hif-1α* mRNA and Hif-1α protein expressions showed dramatic increases with statistical significances [11]. In addition, we found a significant reduction in neovascularization in the choroid in neural retina specific-*Hif-1α* conditional knockout mice in comparison with that in control mice [29]. In this study, we demonstrated that Hif-1α in retinal neuronal cells could be responsive to hypoxic stress but Hif-2α could not. Taken together, this implies that a Hif-1α/VEGF axis in retinal neurons may be a main regulator for ocular neovascularization. However, another group suggested that VEGF and HIF-2α in astrocytes may be essential for pathological neovascularization in their OIR model [47]. The other group demonstrated that hematopoietic HIF-2α deficiency could reduce pathological neovascularization through the modulation of endothelial cell death [48]. Therefore, we think that more comprehensive studies regarding HIF subtypes as well as VEGF-producing cell types and systemic vascular cell conditions may be needed for a better understanding of pathological mechanisms for ocular neovascularization.

Hypoxia-responsive genes other than *Vegf* were also examined in this study. Interestingly, *Bnip3* expression was not dramatically reduced by AHX treatment while *Vegf* and *Pdk1* expressions showed the expected results. BNIP3 is a membrane-associated protein that is primarily localized to mitochondria [49]. BNIP3 expression is induced by hypoxia and is closely related to hypoxia-induced apoptosis in various cell types [50]. However, the regulation of BNIP3 induction by Hif-1α is still under investigation. A previous study reported that treatment with an inhibitor of the mitogen-activated protein kinase (MAPK) pathway inhibited the activation of Hif-1α and its downstream target genes [51]. However, this inhibitor had a less reductive effect on BNIP3 expression in primary mouse hepatocytes [52]. Another study showed that hypoxia-induced upregulation of BNIP3 was not dependent on HIF-1α activation [53]. In our study, even though *Bnip3* expression was not significantly reduced by AHX treatment, we could find a decreasing tendency of its expression. Taken together, we consider that contributions of BNIP3 induction by HIF-1α activation under hypoxic conditions could vary depending on cell types. This could be one of the reasons why AHX treatment dramatically suppressed hypoxia-responsive genes such as *Vegf* and *Pdk1*, but not *Bnip3*.

To date, anti-VEGF drugs are the main pharmacological approach for neovascularization in retinal degeneration [5]. However, long-term VEGF therapies may induce photoreceptor cell atrophy [54]. This is because long-term VEGF administration may suppress the physiological amount of VEGF, which is essential for the normal function of retinal cells [55]. Based on our data, AHX may minimize the alteration of a physiological amount of VEGF, only targeting pathological HIF activation to reduce a pathological amount of VEGF. In addition, our examination for test material-related toxicity demonstrated that repetitive administrations of AHX did not change the body weights of the administered mice. Taken together, this implies that AHX may work on pathological conditions, and a long-term AHX therapy approach with safety could be applicable. However, more detailed testing for unexpected adverse effects of AHX needs to be undertaken for future practical uses.

Current anti-VEGF agents have a high cost for chronic treatments. In addition, the method of administration is invasive. However, AHX can be easily and less-expensively synthesized [56] as it endogenously exists in many crops including rice, wheat, corn, potato and so on [57,58], and an oral administration of AHX is accessible, which is safer, easier to treat, patient-friendly and pain-free [59]. However, direct comparison analyses regarding the safety and effectiveness between these approaches may need to be performed to increase our understanding.

## 5. Conclusions

In conclusion, we screened compounds that originated from mushroom-forming fungi as prospective HIF inhibitors and demonstrated that AHX had a suppressive effect against pathological retinal neovascularization in the murine OIR model. Furthermore, AHX suppressed upregulated hypoxia-responsive gene expressions, especially VEGF. AHX could be useful as a promising drug for neovascularization in ocular diseases.

## 6. Patents

The data in the current research are under consideration for a patent (application no. 2020-103954).

## Figures and Tables

**Table 1:** Primer list.

Name	Direction	Sequence (5′ → 3′)	Accession Number
*Hprt*	Forward	TCAGTCAACGGGGGACATAAA	NM_013556.2
	Reverse	GGGGCTGTACTGCTTAACCAG	
*Hif-1α*	Forward	GGTTCCAGCAGACCCAGTTA	NM_001313919.1
	Reverse	AGGCTCCTTGGATGAGCTTT	
*Hif-2α*	Forward	CTGAGGAAGGAGAAATCCCGT	NM_010137.3
	Reverse	TGTGTCCGAAGGAAGCTGATG	
*Vegf*	Forward	CCTGGTGGACATCTTCCAGGAGTACC	AY707864.1
	Reverse	GAAGCTCATCTCTCCTATGTGCTGGC	
*Bnip3*	Forward	GCTCCCAGACACCACAAGAT	NM_009760.4
	Reverse	TGAGAGTAGCTGTGCGCTTC	
*Pdk1*	Forward	GGCGGCTTTGTGATTTGTAT	NM_172665.5
	Reverse	ACCTGAATCGGGGGATAAAC	

**Table 2:** Screenings of hypoxia-inducible factor (HIF) inhibitors from mushrooms.

Name	1st Trial in 3T3		2nd Trial in ARPE-19		3rd Trial in 661W	
	Fold Change ± SD	*p*-Value	Fold Change ± SD	*p*-Value	Fold Change ± SD	*p*-Value
Topotecan	0.96 ± 0.26	0.855	0.84 ± 0.01	0.002 **	0.81 ± 0.11	0.074
Doxorubicin	0.30 ± 0.06	0.014 *	1.10 ± 0.09	0.135	0.71 ± 0.05	0.006 **
**AHX**	0.39 ± 0.06	0.021 *	0.39 ± 0.03	<0.001 ***	0.42 ± 0.07	0.001 **
**AOH**	0.35 ± 0.03	0.016 *	0.93 ± 0.04	0.091	0.85 ± 0.08	0.086
ICA	1.02 ± 0.32	0.939				
Erinacine A	1.50 ± 0.16	0.054				
Hericenone C	1.25 ± 0.11	0.224				
Hericenone D	0.79 ± 0.04	0.276				
Hericenone E	0.67 ± 0.04	0.115				
Grifolin	0.76 ± 0.06	0.223				
Negrifolin	0.82 ± 0.03	0.338				

The fold change in HIF activity was compared with the value of CoCl_2_-induced HIF activity. Statistically significant inhibitory effects of compounds from mushrooms are shown in bold typeface (* *p* < 0.05, ** *p* < 0.01, *** *p* < 0.001). AHX: 2-azahypoxanthine; AOH: 2-aza-8-oxo-hypoxanthine; ICA: midazole-4-carboxamide.
